# Kongresse der deutschen und österreichischen Gesellschaft für Pneumologie 2025: Highlights aus dem Bereich der Interventionellen Pneumologie

**DOI:** 10.1055/a-2790-3523

**Published:** 2026-02-24

**Authors:** Eldridge Limen, Georg Murauer, Michael Meilinger, Ralf-Harto Hübner, Judith Brock, Daniela Gompelmann

**Affiliations:** 1 14996 Abteilung für Pneumologie und Beatmungsmedizin, Thoraxklinik am Universitätsklinikum Heidelberg, Heidelberg, Deutschland; 2 31415 Translational Lung Research Center Heidelberg (TLRC), German Center for Lung Research (DZL), Heidelberg, Germany; 3 553088 Abteilung für Innere Medizin 4, Pneumologie und Infektiologie, Kepler-Universitätsklinikum Linz, Linz, Österreich; 4 14903 Abteilung für Innere Medizin und Pneumologie, Krankenhaus Nord, Wien, Österreich; 5 27271 Klinik für Pneumologie, Beatmungsmedizin und Intensivmedizin mit dem Arbeitsbereich Schlafmedizin, Charité – Universitätsmedizin Berlin, Berlin, Deutschland; 6 Universitätsklinik für Innere Medizin II, Medizinische Universität Wien, Wien, Österreich; 7 Comprehensive Cancer Center, Medizinische Universität Wien, Wien, Österreich

**Keywords:** interventionelle Pneumologie, Rundherd, Emphysem, interventional pulmonology, pulmonary nodule, emphysema

## Abstract

Dieser Artikel fasst die Highlights im Bereich der interventionellen Pneumologie der Kongresse der deutschen Gesellschaft für Pneumologie (DGP) im April 2025 und der österreichischen Gesellschaft für Pneumologie (ÖGP) im Oktober 2025 zusammen. In inspirierenden und abwechslungsreichen Kongressprogrammen wurden zahlreiche prospektive und retrospektive Studien vorgestellt, die wertvolle Erkenntnisse im Bereich der interventionellen Pneumologie geliefert und neue Perspektiven eröffnet haben. Wesentliche Themen umfassten Innovationen im Bereich der endoskopischen Rundherddiagnostik und -therapie sowie neue Entwicklungen im Gebiet der endoskopischen Lungenvolumenreduktion.

## Abstract (en)

This article summarises the highlights in the field of interventional pneumology from the congresses of the German Society of Pneumology (DGP) in March 2025 and the Austrian Society of Pneumology (ÖGP) in October 2025. Numerous prospective and retrospective studies were presented in inspiring and varied congress programmes, providing valuable insights into the field of interventional pneumology and discussing new perspectives. Key topics included innovations in the diagnostic and therapeutic bronchoscopy for peripheral pulmonary nodules as well as new developments in the field of endoscopic lung volume reduction.

1

Im Rahmen der Kongresse der Deutschen Gesellschaft für Pneumologie (DGP) im April 2025 und der Österreichischen Gesellschaft für Pneumologie (ÖGP) im Oktober 2025 trafen sich zahlreiche Pneumolog*innen aus Deutschland und Österreich, um sich über aktuelle Entwicklungen in der Pneumologie auszutauschen. Die Teilnehmer*innen präsentierten neue Studienergebnisse und profitierten von einem abwechslungsreichen und informativen Veranstaltungsprogramm.

Auf dem Gebiet der interventionellen Pneumologie wurden neue bronchoskopische Verfahren präsentiert und aktuelle Studienergebnisse intensiv diskutiert. Themenschwerpunkte waren u.a. Sedierungstechniken in der Bronchoskopie, navigationsgestützte und robotisch-assistierte Bronchoskopie zur Sicherung peripherer Lungenrundherde, neue Erkenntnisse im Bereich der endoskopischen Lungenvolumenreduktion sowie Innovationen für eine schnellere zytologische Diagnostik. Dieser Artikel fasst die wichtigsten Highlights aus dem Bereich der interventionellen Pneumologie der DGP- und ÖGP-Kongresse 2025 zusammen.

## Sedierung in der Bronchoskopie

Sedierungsverfahren spielen in der Bronchoskopie eine zentrale Rolle, um sowohl den Patientenkomfort als auch die Patientensicherheit zu gewährleisten und gleichzeitig ein optimales Atemwegsmanagement zu ermöglichen. Die Wahl des geeigneten Sedierungsverfahrens hängt maßgeblich von der Komplexität des Eingriffs, der Eingriffsdauer, der Erfahrung des Untersuchers, den Komorbiditäten der Patient*innen sowie von den verfügbaren Ressourcen und den entstehenden Kosten ab 1 . I.d.R. stellt die moderate bis tiefe Sedierung bis hin zur Allgemeinanästhesie den Standard bei Interventionen dar. Die Durchführung der Sedierung ist eine ärztliche Aufgabe, die von einer entsprechend qualifizierten Ärztin oder einem Arzt verantwortet werden muss. Dies kann im Fall einer Sedierung der/die Bronchoskopierende mit Intensiverfahrung sein, sofern eine zweite Person für die Applikation der Sedierung und die Kontrolle der Vitalparameter anwesend ist. Die Applikation der Sedierung kann dabei an speziell ausgebildetes Pflegepersonal (sog. NAPS, Nurse-Administered Propofol Sedation) delegiert werden, welches angesichts des aktuellen Ärztemangels zunehmend an Bedeutung gewinnt.

In einer auf dem DGP 2025 vorgestellten systematischen Übersichtsarbeit von Matthes et al., basierend auf 127 Studien mit insgesamt 38.664 bronchoskopischen Untersuchungen, konnte gezeigt werden, dass die vom Pflegepersonal durchgeführte Propofol-Sedierung im Vergleich zur ärztlich geführten Sedierung hinsichtlich des primären Endpunkts der Mortalität keinen signifikanten Unterschied aufwies (Mortalität NAPS 0,0% vs. Arzt 0,0%; p-Wert 1,00). Es traten insgesamt 4 Todesfälle in der NAPS-Kohorte auf, davon 3 möglicherweise oder wahrscheinlich sedierungsbedingt. Interessanterweise traten Unterschiede im Umgang mit kardiopulmonalen Ereignissen zwischen den beiden Gruppen auf. Während in der ärztlich geführten Gruppe vermehrt unmittelbare kardiopulmonale Interventionen im Untersuchungsraum durchgeführt wurden (z.B. die Gabe von Vasopressoren, Flumazenil oder Naloxon sowie manuelle oder maschinelle Beatmung), reagierte die NAPS-Gruppe häufiger mit einer intensivmedizinischen Verlegung: Die Rate kardiopulmonaler Ereignisse war in der NAPS-Gruppe signifikant niedriger (0,65 vs. 1,16%; p-Wert <0,001), wohingegen die Rate intensivstationärer Verlegungen signifikant höher ausfiel (0,35 vs. 0%; p-Wert <0,001). Die Gabe von Propofol allein hatte keinen Einfluss auf die Mortalität 2 .

Diese Ergebnisse werden durch eine retrospektive Analyse von Boschung et al. am Klinikum Solingen gestützt, in der hinsichtlich der Komplikationsrate kein signifikanter Unterschied zwischen der NAPS-Gruppe und der klassisch ärztlich geführten Sedierung festgestellt wurde (3,0 vs. 2,4%; p-Wert 0,529) 3 . Das Pflegepersonal absolvierte dabei eine strukturierte, ärztlich supervidierte Einarbeitung über 6 Monate sowie einen Sedierungskurs der Deutschen Gesellschaft für Endoskopieassistenz (DEGEA). Auch der Einsatz von Propofol war in der NAPS-Gruppe nicht mit einem erhöhten Komplikationsrisiko assoziiert (2,5 vs. 2,1%; p=0,443) 3 . Die aktualisierte Leitlinie der Deutschen Gesellschaft für Pneumologie und Beatmungsmedizin (DGP) hat ebenfalls Stellung zu diesem Thema genommen. Die Sicherheit und Machbarkeit einer nicht-anästhesiologisch ärztlich oder durch Pflegepersonal geführten Sedierung mit oder auch ohne Propofol konnten in mehreren Studien bereits bestätigt werden 4 .

Bei einer längeren Verfahrensdauer sowie bei komplexen Untersuchungen sollte eine adäquate Atemwegssicherung gewährleistet sein. Diese kann sowohl subglottisch (starres Bronchoskop, Endotrachealtubus) als auch supraglottisch (Larynxmaske) erfolgen. Der Einsatz einer Larynxmaske ermöglicht insbesondere die Inspektion weit proximaler Trachealläsionen. Geissmann et al. untersuchten die Sicherheit der Hochfrequenz-Jetventilation (HFJV) über die Larynxmaske. In dieser retrospektiven Analyse, die 326 Patient*innen (überwiegend mit einem ASA-Score von IV) umfasste, lag die 30-Tage-Mortalitätsrate bei lediglich 0,6%. Die häufigsten Komplikationen waren eine kurzfristige hämodynamische Instabilität (12%), intermittierende Hypoxämie (11%) sowie eine vorübergehende Notwendigkeit einer nicht-invasiven Beatmung (14%). Eine intensivmedizinische Aufnahme war nur bei 1,5% der Patient*innen erforderlich. Auch die Aspirationsrate war mit 1,8% insgesamt niedrig. Das in Donaustauf seit Jahren etablierte Verfahren mit Larynxmaske und HFJV zeigt abschließend ein gutes Machbarkeits- und Sicherheitsprofil 5 .

### Sicherheit der Bronchoskopie bei Hochrisikopatient*innen

Die Durchführung einer Bronchoskopie ist bei bestimmten Patientengruppen mit einem erhöhten Risiko assoziiert und bedarf einer präzisen Benefit-Risiko-Abwägung 6 7 . Merza et al. untersuchten in einer monozentrischen retrospektiven Analyse die Sicherheit der Bronchoskopie bei 63 Patient*innen mit Karzinomen im Hals-Nase-Ohr (HNO)-Bereich und stellten die Ergebnisse auf dem ÖGP 2025 vor 8 . Bei Patient*innen mit HNO-Tumoren können Faktoren wie Schwellungen, vulnerables Gewebe, eingeschränkte Mundöffnung, Stimmlippenparesen oder Kontrakturen im Pharynx-/Larynxbereich zu erschwerten Bedingungen bei der Bronchoskopie führen. Von insgesamt 73 Bronchoskopien bei 63 Patient*innen wurden 22 der Bronchoskopien in Sedierung und 51 in Allgemeinanästhesie (Larynxmaske n=19, endotrachealer Tubus n=13, starre Bronchoskopie n=2, Tracheostomie n=17) durchgeführt. Es erfolgten 73 Lavagen, 17 endobronchiale Biopsien, 13 transbronchiale Biopsien (TBB) und 33 endobronchial ultraschallgesteuerte transbronchiale Nadelaspirationen (EBUS-TBNA). Bei insgesamt 73 Prozeduren kam es zu 2 schwerwiegenden Komplikationen. Bei einem/r Patient*in kam es nach einer Entfernung eines Trachealstents, der bei einer malignen Atemwegsstenose implantiert worden war, zur respiratorischen Dekompensation bei Stimmlippenparese mit konsekutiver Notwendigkeit einer Tracheotomie. Bei einem/r weiteren Patient*in musste die Bronchoskopie, die unter Sedierung erfolgte, aufgrund einer respiratorischen Dekompensation abgebrochen werden. Insgesamt ist die Bronchoskopie bei Patient*innen mit HNO-Tumoren eine sichere Prozedur, bedarf jedoch einer interdisziplinären Risiko-Nutzen-Abwägung. Bei zu erwartendem schwierigem Atemweg sollte eine fiberoptische Wachintubation erfolgen.

### Lungenrundherde: Diagnostik und Therapie

Durch die steigende Anzahl inzidentell in Computertomografien des Thorax gefundenen Lungenrundherden 9 sowie in Erwartung der flächendeckenden Einführung des Lungenkarzinom-Screeningprogramms und damit anzunehmender Zunahme pulmonaler Rundherde spielte das Thema der bronchoskopischen Sicherung der Lungenrundherde weiterhin eine zentrale Rolle auf beiden Kongressen.

Büscher et al. untersuchten in einer retrospektiven Analyse mit multivariabler Regression prä- und intraprozedurale Prädiktoren für die diagnostische Ausbeute der virtuellen Navigationsbronchoskopie und präsentierten die Ergebnisse auf dem DGP 2025 10 . In der Analyse, die 260 Patient*innen umfasste, konnten 5 signifikante Prädiktoren identifiziert werden. Positiv assoziiert mit einer erfolgreichen Navigation und Diagnosesicherung waren das Erreichen des virtuellen Zielpunkts in der augmentierten Durchleuchtung, das Vorliegen eines mindestens semizirkulären radialen endobronchialen Ultraschall (EBUS)-Signals sowie das Vorhandensein eines positiven Bronchuszeichens. Negativ beeinflusst wurden hingegen die Erfolgsraten der Navigationsbronchoskopie durch eine große Distanz zwischen Eintrittspunkt und Läsion sowie durch eine hohe Anzahl an Atemwegsverzweigungen entlang des geplanten Navigationspfads.

Bei Läsionen ohne nachweisbarem Bronchuszeichen kann die transparenchymale noduläre Zugangstechnik (Bronchoscopic Transparenchymal Nodule Access, BTPNA) einen verbesserten Zugang ermöglichen und sowohl die diagnostische Trefferquote als auch die diagnostische Ausbeute erhöhen. Büscher et al. präsentierten auf dem DGP-Kongress 2025 eine modifizierte Form der TPNA („transparenchymal nodule access“)-Technik, bezeichnet als „Essen Tunnel“ 11 . Nach Identifikation der Zielläsion mittels Navigationsbronchoskopie wurde ein transparenchymaler Zugang durch gezielte Penetration der Bronchuswand in das Lungenparenchym und anschließende Ballondilatation geschaffen. Durch diesen Zugang konnten eine radiale Ultraschallsonde über ein ultradünnes Bronchoskop direkt zur Läsion vorgeschoben werden. In der retrospektiven Analyse von 37 Patient*innen zeigte sich durch die Tunneltechnik eine signifikante Verbesserung des EBUS-Signals (mit Tunnel 61,3% vs. ohne Tunnel 19,4%; p-Wert <0,01). Die diagnostische Ausbeute bei Läsionen mit zirkulärem EBUS-Signal lag bei 83,3%. Zu beachten ist jedoch das Komplikationsprofil: In 11% der Fälle (n=4) trat ein Pneumothorax auf, und in 2,7% der Fälle (n=1) kam es zu einer moderaten Blutung.

Ein weiteres Thema auf dem DGP-Kongress 2025 waren neue bronchoskopische Therapieansätze beim pulmonalen Frühkarzinom. Sondermann et al. präsentierten Daten aus einer retrospektiven monozentrischen Studie zur Wirksamkeit und zum Sicherheitsprofil der photodynamischen Therapie (PDT) beim endobronchialen Frühkarzinom bei insgesamt 64 Läsionen in 37 Patient*innen 12 . Im Rahmen der PDT erfolgte zunächst die systemische Applikation eines Photosensibilisators (Chlorin, Photofrin oder Foscan), der sich bevorzugt im Tumorgewebe anreichert. Anschließend wurde bronchoskopisch durch gezielte Laserstimulation eine lokale Gewebsdestruktion induziert. Dabei zeigte sich Chlorin als die Substanz mit dem besten Behandlungsergebnis und gleichzeitig dem günstigsten Nebenwirkungsprofil. Zu den häufigsten Nebenwirkungen zählten Fibrinausschwitzungen (89,4%), die in 39,5% der Fälle zu einem kompletten Verschluss des nachgeschalteten Bronchus führten, sowie postinterventionelle Atemwegsinfektionen (32,4%). Die Tumorgröße hatte einen signifikanten Einfluss auf die Remissionsrate: Oberflächliche Läsionen wiesen tendenziell ein längeres krankheitsfreies Überleben auf als polypoide Läsionen. Der Stellenwert dieses Verfahrens muss jedoch insbesondere im Hinblick auf die Wirtschaftlichkeit und die Langzeitergebnisse weiter evaluiert werden.

Ein weiteres innovatives Verfahren zur Behandlung größerer, lokal begrenzter Lungenkarzinome bei primär nicht operablen oder bestrahlbaren Patient*innen stellt die robotisch-assistierte Mikrowellenablation (MWA) dar. Dieses Verfahren wurde bislang v.a. als perkutane, CT-gesteuerte Technik angewendet. Brock et al. präsentierten eine Fallserie einer prospektiven monozentrischen Treat-and-Resect-Studie zur bronchoskopischen Mikrowellenablation unter Verwendung der robotisch-assistierten Bronchoskopie (RAB) und des Cone-Beam-CT (CBCT) 13 . Eingeschlossen wurden Patient*innen mit Tumoren im Stadium I–IIA ohne Hinweise auf Lymphknotenmetastasen sowie mit Tumorlokalisationen jenseits der dritten Bronchialgeneration. Mithilfe der robotisch-assistierten Navigation konnten die peripher gelegenen Läsionen gezielt erreicht und die Mikrowellensonde in der Tumorregion platziert werden. Die exakte Positionierung der Sonde („Tool-in-Lesion”) wurde durch Cone-Beam-CT-Bildgebung bestätigt. Die Dauer der Ablation richtete sich nach der Größe der Läsion. Im Anschluss war eine definitive Tumorresektion vorgesehen, um die histopathologischen Veränderungen sowie den therapeutischen Effekt der Mikrowellenablation weiter zu untersuchen. In den vorgestellten 3 untersuchten Fällen erwies sich das Verfahren als technisch gut durchführbar. Die postinterventionellen Komplikationen entsprachen dem aus der bisherigen Literatur bekannten Sicherheitsprofil mit führend Pneumonitis und Pleuritis. Die Studie wurde aufgrund schwieriger Rekrutierungsbedingungen nach 3 Patient*innen beendet.

### Endoskopische Lungenvolumenreduktion

Im Rahmen des DGP- als auch des ÖGP-Kongresses wurden neue Studiendaten zur endoskopischen Lungenvolumenreduktion (ELVR) bei Patient*innen mit chronisch obstruktiver Lungenerkrankung (COPD) und schwergradigem Emphysem vorgestellt.

Die älteste und mittlerweile im klinischen Alltag etablierte ELVR-Methode ist die endoskopische Ventilimplantation, die zur Atelektase des am meisten von dem Emphysem betroffenen Lungenlappens und somit zur lobären Volumenreduktion führt. Die häufigste Komplikation nach Ventilimplantation stellt die Ausbildung eines Pneumothorax dar. Brock et al. präsentierten retrospektive, monozentrische Daten von 532 Patient*innen mit Pneumothorax nach Ventilimplantation und erhielten hierfür den klinischen DGP-Forschungspreis 14 . In der Studie wurde der Pneumothorax anhand der klinischen Konsequenzen in 3 Schweregrade klassifiziert: leicht – kein Bedarf einer Thoraxdrainage, mittelschwer – Einlage einer Thoraxdrainage, schwer – Einlage einer Thoraxdrainage mit nachfolgender Explantation der Ventile. Die Gesamtrate der Pneumothoraces betrug 19%. Dabei trat der Pneumothorax signifikant häufiger nach einer Ventilimplantation in die Oberlappen (31,3%) als in die Unterlappen (11,3%; p-Wert <0,001) auf, insbesondere die Ventilimplantation in den linken Oberlappen war mit einem hohen Pneumothoraxrisiko assoziiert. Von den beobachteten Pneumothoraces waren 30,4% leicht, 30,4% mittelschwer und 39,2% schwer. Diese Ergebnisse unterstreichen die Bedeutung einer sorgfältigen individuellen Risiko-Nutzen-Abwägung bei der Indikationsstellung zur Ventilimplantation, insbesondere in Abhängigkeit vom Ziellappen und der Integrität der interlobären Fissuren. Eine interdisziplinäre Aufklärung und Risikobewertung sind daher essenziell.

Eine weitere relevante Komplikation nach endobronchialer Ventilimplantation ist die postinterventionelle Atemwegsinfektion. Die Praxis einer peri- und postinterventionellen antibiotischen Prophylaxe hat sich in der klinischen Routine etabliert, obwohl es keine randomisierte Studie dazu gibt. Pappe et al. präsentierten im Rahmen des DGP-Kongresses 2025 eine retrospektive Analyse aus dem deutschen Lungenemphysemregister sowie Daten aus der Charité – Universitätsmedizin Berlin 15 . Insgesamt wurden 900 Patient*innen mit Ventilimplantation eingeschlossen und in 3 Gruppen unterteilt: einmalige periinterventionelle antibiotische Gabe, mehrtägige antibiotische Gabe, keine antibiotische Prophylaxe. Der retrospektiven Natur der Analyse bleibt die Frage geschuldet, nach welcher Maßgabe die unterschiedlichen Antibiotikaregime ausgewählt werden und ob hier ein Selektionsbias vorliegt. Interessanterweise zeigte sich kein signifikanter Unterschied in der Rate postinterventioneller Pneumonien (p-Wert 0,087) oder COPD-Exazerbationen (p-Wert 0,117) innerhalb der ersten 3 Monate nach der Intervention zwischen den Gruppen. Zudem war die mehrtägige antibiotische Prophylaxe mit einer höheren Inzidenz unerwünschter Arzneimittelwirkungen assoziiert, insbesondere Diarrhö und kutane Nebenwirkungen wie Exantheme. Diese Ergebnisse werfen kritische Fragen zur Evidenzbasis der aktuell etablierten Prophylaxestrategien auf und unterstreichen die Notwendigkeit einer prospektiven, randomisierten kontrollierten Studie zur Klärung der Effektivität und Sicherheit der periinterventionellen Antibiotikagabe bei Ventilimplantation.

Ein weiteres relevantes Thema beim diesjährigen Kongress der DGP war auch die ELVR bei geriatrischen Patient*innen. Hintergrund ist, dass randomisierte, kontrollierte Studien zur ELVR bislang eine obere Altersgrenze von 75 Jahren vorsahen, wodurch ältere Patientenkollektive unterrepräsentiert sind. Eisenmann et al. präsentierten eine retrospektive Analyse aus dem deutschen Lungenemphysemregister, in das 360 Patient*innen mit Ventilimplantation eingeschlossen wurden 16 . Die Kohorte wurde in 2 Altersgruppen unterteilt: <70 Jahre und ≥70 Jahre. Bereits in den Baselinedaten zeigten sich signifikante Unterschiede: Patient*innen ≥70 Jahre wiesen eine signifikant bessere Lungenfunktion auf, jedoch eine deutlich höhere kardiovaskuläre Komorbiditätsrate sowie eine geringere Leistungsfähigkeit im 6-Minuten-Gehtest. Hinsichtlich des postinterventionellen Outcomes zeigten sich jedoch keine signifikanten Unterschiede in der Verbesserung der Lungenfunktion, der Symptomlast oder der Belastbarkeit zwischen den beiden Altersgruppen. Allerdings trat in der Gruppe der ≥70-Jährigen signifikant häufiger ein Pneumothorax als Komplikation auf (14,2 vs. 7,5%; p-Wert <0,044). Eine weitergehende Risikoanalyse, bspw. in Bezug auf die Emphysemverteilung, wurde nicht durchgeführt, sodass die Frage nach der Ursache für die erhöhte Pneumothoraxrate offenbleibt. Insgesamt zeigt diese Studie, dass auch bei älteren Patient*innen eine ELVR mittels Ventilimplantation effektiv und sicher durchgeführt werden kann, wenngleich mit einer erhöhten Komplikationsrate, insbesondere hinsichtlich Pneumothorax, zu rechnen ist. Diese Ergebnisse sprechen gegen eine pauschale Altersgrenze und unterstützen eine individualisierte Risiko-Nutzen-Abwägung bei geriatrischen Patient*innen.

Ein weiteres Verfahren zur bronchoskopischen Lungenvolumenreduktion, welches insbesondere bei apikal betontem Lungenemphysem mit inkompletten Fissuren Verwendung findet, stellt die bronchoskopische thermische Dampfablation (BTVA) dar, bei der durch die Instillation von heißem Wasserdampf eine inflammatorische Reaktion und dadurch eine Schrumpfung erzielt wird. Kontogianni et al. stellten die Daten aus dem internationalen, multizentrischen Register zur BTVA mit Folge-Untersuchungen nach 6 und 12 Monaten vor 17 . In die aktuelle frühe Auswertung wurden 249 Patient*innen, die eine BTVA erhalten hatten, eingeschlossen. Die Daten zeigen eine moderate, über 12 Monate anhaltende Reduktion des Residualvolumens (RV) um –458 ml (p-Wert <0,001). Auch in der gesundheitsbezogenen Lebensqualität, gemessen mittels des St. George’s Respiratory Questionnaire (SGRQ), wurde eine signifikante Verbesserung mit einer mittleren Punktedifferenz von 5,5±16,7 Punkte (p-Wert <0,001) beobachtet. Im Rahmen einer Subanalyse wurden die Patient*innen anhand ihres Therapieansprechens klassifiziert: „General Responders“ waren definiert als Patient*innen mit einer anhaltenden Verbesserung von entweder FEV _1_ oder RV über 12 Monate, „Super Responders“ zeigten Verbesserungen in beiden Parametern. Erste Daten deuten darauf hin, dass ein höheres Ausgangs-RV sowie eine erhöhte funktionelle Residualkapazität (FRV) mit einem besseren Ansprechen assoziiert sind. Die COVID-19-Pandemie führte zu einer Reduktion der verfügbaren Follow-up-Daten, sodass eine abschließende Bewertung des Gesamtregisters zum jetzigen Zeitpunkt noch aussteht. Dennoch zeigen die vorläufigen Ergebnisse, dass BTVA eine vielversprechende Option zur ELVR bei geeigneten Patient*innen darstellen kann.

Im Rahmen des internationalen BTVA-Registers analysierten Trudzinski et al. retrospektiv die Rolle des Geschlechts im Hinblick auf das lungenfunktionelle Ansprechen nach BTVA 18 . Insgesamt wurden 249 Patient*innen ausgewertet, darunter 116 Frauen und 133 Männer. Die Gesamtkohorte zeigte ein moderates Ansprechen auf die Therapie hinsichtlich Lungenfunktion und Lebensqualität bis zu 12 Monate nach der Intervention. Dieses Ansprechen war jedoch signifikant durch die Frauengruppe geprägt, die ein besseres Ansprechen aufwies. Das stärkere Ansprechen bei Frauen könnte durch eine ausgeprägtere inflammatorische Reaktion und somit stärkere fibrotische Umbauprozesse erklärt werden, was letztlich zu einer effektiveren Volumenreduktion führt. Diese Hypothese bedarf jedoch weiterer Untersuchungen.

Zur Charakterisierung der inflammatorischen Reaktion nach BTVA untersuchten Pladeck et al. das radiologische Erscheinungsbild mittels Computertomografie etwa 6 Wochen postinterventionell in einer Fallserie von 9 Patient*innen 19 . Dabei konnten 2 unterschiedliche Inflammationsmuster identifiziert werden: pneumonitisches Muster – ausgeprägte parenchymatöse Infiltrationen, die mit einer stärkeren Volumenreduktion, gemessen durch CT-basierte Marker wie das Emphysemvolumen, assoziiert waren; peribronchitisches Muster – umschriebene Verdichtungen entlang der Bronchien mit geringer Auswirkung auf das Emphysemvolumen. Interessanterweise zeigte sich im Verlauf kein signifikanter Unterschied zwischen den Gruppen hinsichtlich des Residualvolumens oder der totalen Lungenkapazität. Es sind weitere Studien mit größeren Patientenzahlen notwendig, um verlässliche Prädiktoren zu identifizieren und die zugrunde liegenden Mechanismen besser zu verstehen.

Ein neues Verfahren der ELVR stellt die Implantation sog. „Airway Scaffolds“ (BREATHE Airway Scaffold; Apreo Health) dar. Diese aus Nitinol bestehenden helixförmigen Implantate stabilisieren die bei der Exspiration kollabierenden Atemwege der Emphysempatient*innen, verhindern somit den Atemwegskollaps und wirken somit der dynamischen Überblähung entgegen 20 . Klemm et al. untersuchten die Atemwegsdurchmesser vor und 6 Monate nach der Implantation der Airway Scaffolds und stellten diese Daten auf dem ÖGP 2025 vor 21 . Dabei erhoben die Autoren die Atemwegsdurchmesser anhand der CT-Thoraces der Feasibility-Studien BREATHE-1 (Australien) und BREATHE-2 (Europa), in denen bei 60 Patient*innen mit schwerem Lungenemphysem jeweils bis zu 3 Scaffolds pro Lungenflügel implantiert worden waren. Die Atemwegsdurchmesser wurden proximal, mittig und distal der Implantate mittels CT-Thorax vor und nach 6 Monaten gemessen (VIDA Diagnostic, Inc.) und die Anzahl der messbaren Lokalisationen erhoben. Die Anzahl der messbaren Atemwegspositionen erhöhte sich signifikant nach der Implantation der Airway Scaffolds (p<0,0001) und deutet somit auf einen erhöhten Atemwegsdurchmesser hin. Auch die Lungenüberblähung, gemessen am RV nahm signifikant ab (–753±874 ml [p<0,0001]). Weitere prospektive, randomisiert-kontrollierte Studien sind erforderlich, um die Effektivität und die Nebenwirkungen dieser noch neuen Methode zu evaluieren.

### Transbronchiale Kryobiopsie bei interstitiellen Lungenerkrankungen

Die transbronchiale Kryobiopsie ist mittlerweile ein etabliertes Verfahren zur Diagnostik interstitieller Lungenerkrankungen (ILD), wobei bislang standardmäßig 1,7, 1,9- oder 2,4-mm-Kryosonden eingesetzt werden. Fiedler et al. untersuchten die diagnostische Leistungsfähigkeit einer dünneren, 1,1-mm-Kryosonde in der Diagnostik der ILD 22 . In der vorgestellten retrospektiven Analyse der Daten von 26 Patient*innen wurden insgesamt 8 Biopsate aus mindestens 2 Lungenlappen und unterschiedlichen Segmenten entnommen. In 96% der Fälle konnte anhand der entnommenen Proben eine spezifische histopathologische Diagnose gestellt werden. Komplikationen traten in 23% der Fälle in Form von leichten bis moderaten Blutungen auf. Eine schwere Blutung wurde berichtet. Diese vorläufigen Daten deuten darauf hin, dass die 1,1-mm-Kryosonde ein vergleichbares diagnostisches Potenzial wie die herkömmlich verwendeten, dickeren Kryosonden aufweist. Die Blutungskomplikationen lagen im vergleichbaren Bereich zur Standardtechnik. Weitere Studien mit größerer Fallzahl und prospektivem Design sind erforderlich, um diese Fragestellung zu beantworten und eine standardisierte Protokollierung der Anwendung zu ermöglichen.

### Rapid Online Evaluation (ROLE)

Die diagnostische Aussagekraft endoskopisch gewonnener zytologischer Proben ist essenziell zur Beurteilung der Dignität und Ausbreitung maligner Erkrankungen. In der klinischen Praxis ist jedoch häufig eine unzureichende oder nicht eindeutige Probenqualität festzustellen, was zu Folgeeingriffen oder im ungünstigsten Fall zu invasiveren chirurgischen Maßnahmen führen kann. Elmas et al. präsentierten auf dem diesjährigen DGP-Kongress ein innovatives Verfahren zur Rapid Online Evaluation (ROLE), das eine zeitnahe Einschätzung der Probenqualität bereits während der Intervention ermöglicht 23 . Hierzu wurden die gewonnenen Proben unmittelbar nach der Entnahme für 30 Sekunden lang in Methanol fixiert, anschließend mit einer Giemsa-Stammlösung gefärbt und mithilfe eines digitalen Mikroskops (Preci-Point iO:M8) erfasst. Die Bilddaten wurden in Echtzeit an ein pathologisches Institut übermittelt und dort telemedizinisch ausgewertet. Die mittlere Auswertungszeit betrug 100 Sekunden (Range: 11–370 Sekunden). Insgesamt wurden im Rahmen der vorgestellten Analyse 190 maligne, 86 benigne sowie 38 unklare Befunde erhoben. Für die eindeutig beurteilbaren Proben (maligne und benigne) ergaben sich eine Sensitivität von 90,5% und eine Spezifität von 98,5%. Die vorgestellte ROLE-Technologie bietet insbesondere für Einrichtungen ohne eigene zytologische oder pathologische Abteilung eine vielversprechende Option. Darüber hinaus eröffnet sie durch die telemedizinische Anbindung auch Potenziale für den internationalen Einsatz, insbesondere in strukturschwachen oder unterversorgten Regionen und Ländern mit begrenztem Zugang zu Pathologiediensten.

### Innovationen in der Thoraxsonografie

Die sonografische Diagnose eines Pneumothorax basiert auf dem Fehlen eines Pleuragleitens. Alternativ kann auch der Lungenpuls, nämlich die pulssynchrone Pleurabewegung durch die weitergeleitete Herzaktion, herangezogen werden. Dieser wird normalerweise im B- und M-Mode dargestellt. Rieder et al. untersuchten im Rahmen einer „Proof-of-Concept“-Studie bei 20 gesunden Probanden den Nachweis des Lungenpulses mittels Powerdoppler und stellten die Ergebnisse auf dem ÖGP 2025 vor 24 . Die Untersuchung erfolgte standardisiert an 6 Punkten (ventral, lateral, dorsal) und bei 3 Thoraxvolumina (Atemruhelage, vollständige Exspiration und Inspiration) mit Dokumentation von Pleuragleiten und Lungenpuls jeweils im B-Bild, M-Mode und Power-Doppler. Die Auswertung erfolgte verblindet durch einen Pneumologen anhand der Videoaufnahmen. Es zeigte sich eine Spezifität des Power-Dopplers von 100% verglichen mit dem diagnostischen Goldstandard des Pleuragleitens. Der Lungenpuls war mittels Generalized Linear Model im B-Bild und im Power-Doppler gegenüber dem M-Mode deutlich besser darstellbar bei sehr hoher Darstellbarkeit am ventralen Thorax. Die Autoren schlussfolgern, dass die Darstellung des Lungenpulses bei Gesunden mittels Power-Doppler dem M-Mode überlegen ist und die gute Reproduzierbarkeit in den ventralen Thoraxbereichen in Akutsituationen zum Pneumothoraxausschluss hilfreich sein könnte.

### Nachwuchsförderung in der interventionellen Pneumologie

Ein zentrales Anliegen des diesjährigen DGP-Kongresses war die gezielte Förderung des pneumologischen Nachwuchses. In diesem Zusammenhang wurde eine eigene Session mit dem Titel „Karriere und Chancen in der interventionellen Pneumologie“ angeboten. Die Veranstaltung bot einen offenen, inspirierenden und kollegialen Austausch zwischen erfahrenen Fachärzt*innen und Forscher*innen sowie interessierten jungen Kolleg*innen. Im Mittelpunkt standen praxisnahe Themen wie der Einstieg in die interventionelle Pneumologie, Forschungsprojekte und -förderung, Vernetzung und Mentoring-Programme. Durch persönliche Erfahrungsberichte und strukturierte Diskussionsrunden erhielten Nachwuchsmediziner*innen wertvolle Einblicke und motivierende Impulse für ihre eigene Laufbahn. Die Session zeigte eindrucksvoll, wie wichtig strukturelle Unterstützung, frühzeitige Förderung und interdisziplinärer Dialog für eine nachhaltige Entwicklung der nächsten pneumologischen Generation sind.

## Footnotes

1 Geteilte Letztautorenschaft
